# Review on EEG-Based Authentication Technology

**DOI:** 10.1155/2021/5229576

**Published:** 2021-12-24

**Authors:** Shuai Zhang, Lei Sun, Xiuqing Mao, Cuiyun Hu, Peiyuan Liu

**Affiliations:** Information Engineering University, Zhengzhou 450001, China

## Abstract

With the rapid development of brain-computer interface technology, as a new biometric feature, EEG signal has been widely concerned in recent years. The safety of brain-computer interface and the long-term insecurity of biometric authentication have a new solution. This review analyzes the biometrics of EEG signals, and the latest research is involved in the authentication process. This review mainly introduced the method of EEG-based authentication and systematically introduced EEG-based biometric cryptosystems for authentication for the first time. In cryptography, the key is the core basis of authentication in the cryptographic system, and cryptographic technology can effectively improve the security of biometric authentication and protect biometrics. The revocability of EEG-based biometric cryptosystems is an advantage that traditional biometric authentication does not have. Finally, the existing problems and future development directions of identity authentication technology based on EEG signals are proposed, providing a reference for the related studies.

## 1. Introduction

In computer science and cryptography, authentication is defined as the confirmation of a user's claimed identity. Authentication is different from identity recognition. Identity recognition identifies who the user is, while authentication determines whether the user's identity is consistent with that declared in the system. The former identifies the unique identifier corresponding to the user characteristics, while the latter determines whether the user is a legitimate user or an attacker of the system. Authentication includes identification, and it needs to first identify the user's identifier corresponding to the user's characteristics. Authentication is widely used in various security systems (such as access control) and information systems such as computer networks. In the real world, each user has a unique real identity, while in the network, each user needs to have a unique digital identity.

**Figure 1:**
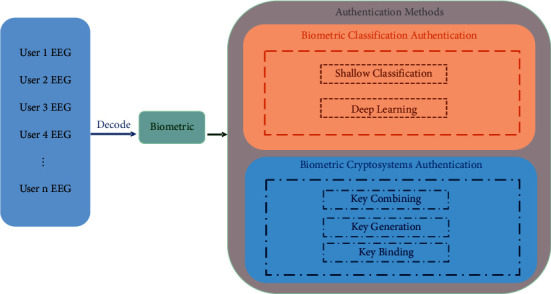
EEG-based authentication methods.

Authentication methods can be divided into three categories: biometric (owned by the individual), password (known to the individual), and token (owned by the individual) [1]. The authentication based on biological features [2] has a wide range of applications. In principle, as long as the physiological or behavioral features of people meet the requirements of universality, uniqueness, stability, and anti-fraud, they can be used as biometric features for authentication, such as the face, fingerprint, iris, voice print, DNA, and gait. However, these features are prone to be tampered with, forged, coerced, and irrevocable. The password-based authentication technology requires that the password should be random and long enough. The password is easy to forget unless the users have a good memory. Also, personal passwords can be stolen, and weak passwords can be attacked with violence and guesswork. The token-based authentication technology, such as digital certificate [3], in which users hold a private key corresponding to their own identity for authentication, is considered the most secure cryptography approach. Real-world tokens such as ID cards and access cards usually require users to carry them around, but they can be copied or stolen. In cryptography, the key is the core basis of authentication in the cryptographic system. In this review, the method of EEG-based authentication can be divided into EEG-based biometric classification authentication and EEG-based biometric cryptosystem authentication. The EEG-based authentication methods are shown in Figure 1.

The electroencephalogram (EEG)-based authentication takes the individual difference in the EEG signals as the only corresponding identity and performs authentication. As a new type of biometric feature [4], EEG signals meet the basic requirements of authentication [5]. Different from the biometric features such as fingerprints and faces, which are easily affected by external factors, EEG signals are relatively stable. Since EEG signals are generated by neural activities in the brain, they have a unique neural pathway pattern and are difficult to forge in terms of physiological theories. Studies have shown that different individuals produce different EEG signals by endogenous spontaneous generation and exogenous stimuli-induced generation [6], confirming the uniqueness of human EEG signals to a certain extent. The EEG signals under stimulus are different from those under a normal condition; that is, EEG signals can be used as a kind of biometric feature to monitor the abnormal state of people. When the brain is in a compulsion state, it cannot produce EEG signals. This phenomenon can be used as an effective indicator to differential between voluntariness and intimidation. Besides, since EEG signals can only be generated by a living body, the EEG signals will no longer exist once an individual dies, making it highly stress-resistant.

Biometric features are generally divided into physiological features (fingerprints, iris, DNA) and behavior features (gait, signature). Because of its high dependence on task behaviors and emotional sensitivity, EEG signals are characterized as a fusion of physiological and behavioral features. Compared with traditional biometric features, task-dependent EEG authentication has the revocability that other biometric authentications do not have. Also, its high dependence on tasks allows it to show different characteristics according to tasks. Once certain characteristics are lost or stolen, they can be revoked in time to prevent the system from being attacked. Therefore, EEG signals can effectively solve the irrevocable problem of traditional biometric features.

The initial research on EEG signals focused on the medical field, especially the abnormal discharge behavior of the brain such as epilepsy and other neurological diseases. With the technological development of the brain-computer interface [7, 8] and the commercial acquisition equipment, the EEG signals can be easily obtained, providing powerful tools for the research on brain neuroscience and promoting the study of EEG signals to a new level. The brain-computer interface establishes a direct interaction path between the brain and the computer. Based on the interface, the brain can directly control the computer or equipment without going through the nerve or the muscle. The brain-computer interface can decode [9] human intentions, mental states, and emotional changes [10] and encode them as control instructions to control external devices [11]. Also, it gives feedback through the neural interface to stimulate and regulate the central nervous system. Although most brain control technologies, intention recognition, and disease diagnosis are hoped to be widely used between different subjects to achieve good compatibility, the decoding of EEG signals of different subjects is actually a kind of decoding of different identities. With the rapid development of consumer-grade noninvasive brain-computer interface devices such as EMOTIV System [12]and NeuroSky [13], the devices have become more portable, commercial, and popular, and they are widely used by researchers. EEG and brain-computer interfaces are faced with security issues [14, 15]. For example, the devices may be attacked, and the private information involved in the personal EEG signals may be leaked [16]. To solve the security problem, authentication can effectively control access, and EEG signals as the biometric feature of the brain-computer interface system are undoubtedly the best natural feature for authentication. Similar to the current smart terminal that can only be used by legitimate users, the EEG and the results of analysis and processing can only be accessed by their owners.

EEG as a biometric has been studied in-depth by many researchers [17]. Gui et al. [17] summarized the EEG as a biometric, and Bidgoly et al. [18] fully discussed the methods and challenges of EEG-based authentication. Based on their work, this review makes a further analysis and proposes to divide the authentication methods into EEG-based biometric classification authentication and EEG-based biometric cryptosystem authentication, which was systematically introduced for the first time. Also, this review analyzes some of the latest research results (such as the influence of EEG frequency channel position on the accuracy of certification, phase synchronization characteristics, and multimodal combination authentication) and safety issues. The rest of this study is organized as follows.  Section 2 introduces the physiological basis of EEG, and Section 3 describes the EEG acquisition methods.  Section 4 introduces the pretreatment methods of EEG biometric authentication.  Section 5 discusses the feature extraction methods of EEG signals.  Section 6 introduces the authentication methods of EEG.  Section 7 presents the existing problems and future development direction of EEG certification. Finally, section 8 concludes the paper.

## 2. EEG

### 2.1. Physiological Characteristics of EEG

EEG is a method of using electrical signals to record brain activity. It is the sum of the postsynaptic potentials of many neurons in the cerebral cortex, and it is a multichannel recording of the subject's response activities in the central and autonomic nervous systems [5]. The central nervous system, including the brain and spinal cord, responds consciously to external stimuli. The autonomic nervous system is a control system that works unconsciously to regulate body functions, such as heart rate, breathing, and pupillary response. EEG signals reflect the neural activities generated by internal and external stimuli. Also, they represent physiological and behavioral information.

EEG contains a large number of physiological characteristics, including individual cognitive ability [19], gender [20], age, disease, emotion [21, 22], and other individual differences. There are considerable individual differences in brain structure and extensive cognitive functions. Different individuals have unique connectivity between different functional areas of the brain [23, 24]. The EEG signal of different individuals performing the same task is significantly different, but that of the same individual performing the same task is relatively stable and repeatable. Therefore, EEG signal has unique and stable characteristics [18]. The EEG is highly dependent on the individual's neural activity that has a very complex and unique nonlinear neural pathway. In this case, it can be affected by external stimuli, personal emotions, pressure, and mental state. The collection of EEG requires special equipment, and the collected information is not exposed like faces or fingerprints, making it difficult for attackers to forge fake faces or use gelatin to forge fingerprints [25].

### 2.2. EEG Frequency

EEG signals have different frequencies in different neural states of brain activity. Researchers are trying to investigate the frequency bands of EEG to find the most suitable one for authentication and reduce the amount of data for analysis. Person in different physical or psychological states displays different EEG frequencies. The rhythmic activity within a certain frequency range represents a specific brain activity condition. Therefore, different frequency bands of EEG are usually studied in a targeted manner, and the characteristics of the bands are listed in Table 1 [17].

Zhang et al. [26] calculated the cosine similarity of the EEG signals of different subjects and revealed that the average similarity of the EEG signal in the delta band is the lowest. That is, the signal contains the most distinguishable features and the most unique information for identification, and it is the most stable waveband in different states. The researchers thought that the better stability of the EEG signal in the delta band is because the delta frequency band is the most basic waveband necessary for physiological activities in any state [26]. Altahat et al. [27], Wang et al. [24], and Li et al. [28] found that the authentication accuracy of the EEG signal in the beta and gamma bands is higher than that of the EEG signals in other frequency bands. The reason is that the EEG signal in the gamma frequency band is the main component of the EEG signals produced by visual information processing tasks, and the EEG signal in the beta frequency band is the main component of the EEG signals produced by visual-related mental tasks. The studies of Kumar et al. [29], Nguyen et al. [30], and Thomas and Vinod [31] have shown that the gamma band has the highest performance. The reason may be that the gamma band is more chaotic and complex than other bands, making it more nonlinear and unpredictable [32].

Although many researchers divide EEG signals into different frequency bands for authentication, the characteristics of individual differences in brain neural activities are distributed in all frequency bands, and there is no single frequency band that can contain all identity-related information [27]. Researchers achieved different results for different frequency bands because they used different stimulus tasks. The main components of EEG signals induced by these stimulation tasks are distributed in different frequency bands, so a high accuracy can be achieved in the frequency band highly corresponding to the task.

### 2.3. Locations of Brain Functional Areas of EEG

The research of Bergson et al. [33] pointed out that the brain has a normal background level, i.e., a spontaneous EEG activity pattern that fluctuates throughout the brain. Human cognition and decision-making have a random effect on this spontaneous EEG background level [33]. Thus, the original EEG is composed of inherent background EEG, task-awareness EEG, and noise. The collection of EEG data generally requires task induction, and the execution of the task corresponds to different specific brain regions. Although the EEG has a high time resolution, its spatial resolution is very low, and it requires the corresponding electrode to be placed in different positions on the scalp. Thus, understanding the area of the brain where the response is generated is crucial to promoting the optimal or suboptimal selection of the number of electrodes used and their positions. The number and positions of the electrodes selected by different researchers for different tasks are listed in Table 2.

Ruiz-Blondet et al. [39] believed that data in the O2 channel are the most stable and effective in the semantically induced ERP paradigm. Jin et al. [40] believed that the characteristics of the identity information are mainly concentrated in the seven channels of Fz, FC1, FC2, Cz, CP1, CP2, and Pz. Salem and Lachiri [41] believed that PO3, PO4, O1, Oz, and O2 carry the most suitable identification information for biometric authentication. Due to individual differences, choosing a different set of EEG channels for each person will improve the accuracy of authentication [27]. Wu et al. [34] built an optimized channel-based model for each user to improve authentication accuracy and robustness. Meanwhile, genetic algorithms can be used to optimize the authentication channel. Therefore, similar to the frequency band selection, the choice of electrode position is also highly dependent on the stimulation task.

## 3. EEG Acquisition on Different Tasks

The neuronal firing of the brain is highly dependent on the human mental state; that is, it is very sensitive to external environmental stimulation and endogenous autonomous regulation. Therefore, it is necessary to design a special collection paradigm to collect EEG signals purposefully. According to whether an EEG is evoked or not, the EEG can be divided into spontaneously generated EEG (such as resting-state potentials and sleep potentials without tasks and stimulation) and evoked EEG with specific stimulus protocols for specific tasks (such as motor imagination and vision for specific imaging tasks) [18], which are used to study EEG signals with temporal characteristics under spontaneous state, environmental perception, and complex cognition. Because of the unique brain neural pathways and thinking patterns between different individuals, there are different types of task signals. Even in the same type of task, the EEG under different tasks is also different. Choosing a suitable induction paradigm will have a great impact on the recognition results, so the choice should be made according to specific tasks.

### 3.1. Resting State

The resting state is a state in which the brain does not perform specific cognitive tasks and remains quiet, relaxed, and awake. As the most basic and essential state of the brain, the resting state does not require subjects to perform specific tasks or receive any external stimuli. Many researchers have proved that the brain of different people in a completely resting state produces different EEG signals, and these signals carry unique characteristics of the subject [36, 42–44]. The EEG is suitable for use in a universal environment. However, this paradigm is greatly influenced by the outside world, and it is difficult to ensure complete silence in a real application environment. However, because of the concealment of EEG, the researcher cannot guarantee that the subject is indeed in a completely quiet state, and the subject's extra activities will seriously affect the accuracy of certification.

### 3.2. Motor Imagination

The motor imaging (MI) induction task induces the corresponding EEG signals when the subject imagines a certain limb or tongue movement. Modern neuroelectrophysiology research reveals that when limb movement or motor imagination is performed, the EEG has significant individual differences [45]. Studies have shown that the resting potential is less noisy than that of the motor imagination task, and the EEG of the motor imagination task is less noisy than that of the real movement task [27]. Since the EEG of motor imagination tasks has better individual differences, it has been used for authentication in recent years [46]. This method is suitable for all kinds of patients with physical disabilities and visual defects, and it has good applicability. However, motor imagination tasks are faced with blindness in some cases, and the execution of motor imagination cannot induce obvious signals.

In recent years, with the development of technology, some imagination tasks similar to the motor imagination paradigm have also been used for authentication. For example, speech imagination is originally applied to people whose brain function still works despite speech muscle damage. However, researchers found that speech imagination is more natural and simpler than motor imagination, and it does not require any external stimulation. Besides, it is relatively difficult to cause fatigue [47].

### 3.3. Event-Related Evoked Tasks

The event-related potential (ERP) evoked task is a special kind of evoked task [48]. It reflects the cognitive process by averaging and superimposing the brain potential recorded from the scalp when a person performs cognitive processing on an object. Event-related potentials have high time resolution and can measure immediate responses to short stimuli. They are usually measured in terms of latency and amplitude changes in the positive and negative potentials in milliseconds after the stimulus occurs. Common event-related potential components can be divided into P100, N100, N200, N250, P300 [35], N400 [49], and so on in order.

The most common event-related potential is the visually evoked potential. Visually evoked tasks mean specific neural activities that are induced by the nervous system and receive external visual stimuli (such as graphics or flashing stimuli) [29]. To generate stable and strong correlation signals, exogenous evoked events are often used as repetitive sensory stimuli, for example, steady-state visually evoked potential (SSVEP). Also, the differences between individuals are obvious. Besides, the SSVEP has a better signal-to-noise ratio and anti-artifact ability. The disadvantage of the visually evoked task is that the task requires a dedicated external device to generate stimulation. Also, the subject should have normal vision, which is not friendly to users with visual problems. Besides, strong visual stimulation is likely to cause visual fatigue and emotional resistance of the subject, affecting the recognition results significantly.

Common ERP evoked tasks include semantic cognition-based neural activity measurement tasks, fatigue driving tasks [50], rapid visual serial representation (RSVP) [51], and steady-state auditory evoked potentials [52]. For example, the task of reading acronyms and mental arithmetic multiplication, as well as some reading tasks, will produce a strong and stable potential of N400 [53, 54]. Event-related tasks such as semantic cognition reflect different responses of the brain to familiar and unfamiliar object stimuli. The potential of N400 can be obtained when subjects are presented with familiar acronyms that are larger than their unfamiliar acronyms [39]. Studies have shown that in the RSVP event-related task, subjects are presented with a continuous image sequence of target and nontarget images, resulting in a strong individual difference in the ERP potential. This difference is widely used for authentication.

### 3.4. Multitask and Task-Independent

It is generally believed that the EEG of one type of task generally has a lower authentication accuracy than that of multiple types. Therefore, some researchers have designed a complex multitask [48] evoked paradigm to make the EEG consist of more individual different feature components, thus achieving higher recognition accuracy. These paradigms include the multitasking paradigm of visually evoked and auditory evoked combination [38], the multitasking paradigm of imaginary text, mathematical operations, and visual stimulation [55], the multitasking paradigm of visual stimulation, multiplication, letter combination, and graphic rotation, and other complex multitasking paradigms [56]. However, data of multitask are more complicated than that of a single task, increasing the difficulty of decoding and also causing mental fatigue and resistance to the subjects. Multitasking can improve the recognition accuracy to a certain extent, and different researchers with different tasks usually use different methods, which makes the method poorly transferable to different tasks. Therefore, some researchers hope that no matter what tasks the subjects are performing, they can achieve a good authentication effect, that is, task-independent [57].

## 4. Data Preprocessing

### 4.1. Time-Domain Analysis

The time-domain analysis method of EEG signal extracts effective information from the time domain of the signal to reduce noise reduction and facilitate further processing. EEG signals are generally manifested as waveforms in the time domain, and the time-domain analysis method can analyze the waveform information. The time-domain waveform of the EEG signal contains all the characteristics of the time dimension. It changes with time and shows the non-stationarity of the signal. Statistical methods and signal smoothing techniques such as mean, median, variance, and normalization are often used to extract the necessary information from the signal to obtain a higher signal-to-noise ratio.

### 4.2. Frequency-Domain Analysis

The frequency-domain feature uses the Fourier transform-based technology to convert the EEG signal into a frequency distribution. The EEG power spectrogram in the frequency domain depends on the change in frequency. According to the power spectrogram, the distribution of different frequency bands of the EEG signal is analyzed. The frequency-domain analysis of EEG signals mainly uses analysis methods such as filters and frequency spectrum estimation. Power spectrum estimation is an analysis method that describes the random characteristics of the EEG signal in the frequency domain. This method is the basis of other frequency-domain analysis methods, such as Chebyshev filter, Butterworth filter, and AR parameter model estimation. Low-pass filtering of EEG signals by the Chebyshev filter can obtain EEG signals of specific frequency bands [34, 58]. The research by Pozo-Banos et al. [48] revealed that the neural features extracted from the EEG spectrum are largely independent of the recorded cognitive tasks and experimental conditions. It is suggested to use this task-independent neural signature for accurate biometric authentication [48].

### 4.3. Time-Frequency Domain Analysis

The time-frequency domain analysis method combines the time-domain and frequency-domain information of EEG signals for analysis. Meanwhile, it transforms the signals of the one-dimensional time dimension and frequency dimension into a two-dimensional form. This method has a few advantages. First, it can avoid the loss of frequency information during time-domain analysis and the loss of signal waveform transients during frequency-domain analysis. Also, it can extract features that cannot be simultaneously expressed in a single domain, such as the frequency information contained in each moment. Currently, the most widely used time-frequency domain analysis methods include short-time Fourier transform, wavelet transform, and wavelet packet decomposition [17].

### 4.4. Spatial-Domain Analysis

To collect enough information for the authentication of EEG signals, researchers generally use multiple electrodes to collect signals, and different tasks involve different brain regions and the associated information between the corresponding electrodes. Spatial-domain analysis can reduce noise, detect and remove artifacts, and analyze the signal components with important characteristics. The commonly used spatial-domain analysis methods include common average reference (CAR) [59], principal component analysis (PCA), independent component analysis (ICA) [38, 60], and Laplacian spatial filter. CAR calculates the average value of all electrodes and eliminates noise by removing the average value of all electrodes. Then, it obtains the most important information in the EEG signal and removes the artifacts in the signal [37].

### 4.5. Nonlinear Dynamics Method

The nonlinear dynamics method analyzes the functional activity state of the brain by combining chaos and fractal theory and other nonlinear dynamics principles and methods. Modern physiology studies believe that the brain is a chaotic dynamic system, and the amplitude exhibits random changes over time [32]. The EEG signal contains the information of thousands of neuron activities. Also, the interconnections and firing behaviors of neurons are nonlinear. Thus, it is considered that the underlying neural subsystem that generates the EEG signal is nonlinear [61]. In recent years, with the study of the correlation between different brain regions and the connectivity of neuron firing, nonlinear methods such as phase synchronization and brain connection networks have been proposed. Researchers focus on nonlinear characteristics such as the firing process and the degree of coordination and asynchrony of brain neurons involved in neural activities. In this way, the information that is involved in the general time-frequency domain can be extracted.

## 5. Feature Extraction

### 5.1. AR Parameter Model

AR parameter model is one of the earliest and most commonly used feature extraction methods for EEG signals [36, 47]. It exploits the Fourier transform-based method to calculate signal spectrum. The key is a linear regression model that combines random variables in the past to describe the random variables in the later period, which usually leads to better results. The spectrum estimation accuracy of the AR model is highly correlated with the model order. If the model order is not high enough, the spectrum estimation will be blurred, and if the model order is too high, the spectrum will exhibit a false peak. The model order depends on the signal spectral content and sampling rate. As early as 1999, Poulos et al. [62] used the fast Fourier transform to preprocess the EEG signal, and they used the signal to construct an 8-order AR model for authentication.

### 5.2. Power Spectral Density

Power spectral density (PSD) defines the distribution of the power of a signal time series with frequency, and it is a measure of the mean square value of a random variable. PSD is used to indicate the distribution of signal power at each frequency point. Although the total energy of a random signal is infinite, its average power is limited. Based on the random characteristics of the EEG signal, the frequency-domain power spectral density of the EEG signal can be analyzed, and the power spectral density characteristics can be extracted for classification or encoding [31].

### 5.3. Common Space Pattern

Common space pattern (CSP) is a spatial-domain filtering feature extraction algorithm for two classification tasks, which can extract the spatial distribution components of each category from multichannel EEG signals. By searching for the projection direction that can best distinguish the two types of signals, the feature vector with a high degree of discrimination can be obtained. Baig et al. [63] firstly used the CSP algorithm to extract the feature set in the state of left-hand and right-hand motion imagination. Then, the feature subset was sent to the SVM classifier to build the classification model. In this way, an average classification accuracy of more than 95% is obtained. However, this algorithm is not suitable for multi-classification problems.

### 5.4. Phase Synchronization

The EEG signal is composed of amplitude and phase information. Due to the lack of phase information measurement and calculation methods, traditional research focuses on the amplitude analysis of a single electrode but ignores the phase information. The cognitive activities in the brain integrate the functions of each brain area and the continuous interaction between the brain areas. Meanwhile, the synchronization in neurobiology adjusts the oscillating neurons to synchronize the discharge of related neurons. Phase synchronization describes the instantaneous phase relationship between different channel pairs, reflecting the interaction between the channel pairs and the difference in the structure of individual white matter. Since phase synchronization is the external manifestation of the individual's intrinsic identity, its measurement can provide a more stable estimation of the brain function connectivity [23] than the time-domain measurement. The brain function connectivity is expressed as the channel position information relationship. Therefore, the relationship between the phase synchronization and the physiological characteristics of the brain can be exploited to analyze the phase synchronization characteristics of each channel. Meanwhile, a phase synchronization matrix or brain connection network [64] can be constructed. Kong et al. [65] regarded each channel as a node in the brain network, and they used the phase synchronization between nodes as the connection strength. The phase synchronization was measured by the phase lock value to generate a connection matrix, and the number of edges connected to the node (i.e., the node degree of the brain network) was synthesized. Based on this, the construction feature was used for authentication [65]. Wang et al. [24] proposed a graph feature analysis method based on the brain-connected network topology. By investigating the interaction mechanism of multichannel brain network structure nodes, a special subjective brain network was defined. Then, the impact of different connection indicators, map characteristics, and EEG signal frequency bands on the performance of biometric recognition was analyzed, revealing the huge individual differences in the brain function connection network constructed by phase synchronization [24].

## 6. Authentication Method

### 6.1. EEG-Based Biometric Classification Authentication

Feature extraction aims to extract the features that can distinguish each individual. The method of classification treats each person as a category, and the extracted features are divided into categories that can correspond to each individual. The classification model is trained through supervised learning to realize a one-to-one correspondence between the features and identities for authentication.

#### 6.1.1. Shallow Classification

*(1) Linear Discriminant Analysis (LDA)*. LDA is a classic linear learning method that tries to find a linear combination of features between different categories to characterize or distinguish them. The samples are projected onto a straight line to make the samples of the same category as close as possible and the samples of different categories as far away as possible. Based on this, classification of the samples can be achieved. LDA is one of the most widely used shallow classifiers in EEG-based authentication [58, 66]. Kong et al. [65] measured the phase synchronization by calculating the phase lock value (PLV). Then, they constructed a coherence matrix to obtain a weighted undirected network and used the node degree of the brain network to generate feature vectors, achieving an accuracy of 95% in the LDA classification. Koike-Akino et al. [35] collected P300 components on the ERP paradigm data set consisting of 25 subjects and extracted features through PCA and partial least squares. Using LDA, an accuracy of 96.7% can be achieved. Seha and Hatzinakos [52] recorded the EEG signals of 40 subjects under the stimulation of multiple auditory tones modulated in the frequency bands of 40 Hz and 80 Hz based on the steady-state auditory evoked task. Then, they used canonical correlation analysis (CCA) to extract features and input them into LDA. In this way, an accuracy classification of 96.46% was achieved.

*(2) Support Vector Machine (SVM)*. SVM is a two-classification model that classifies samples by a hyperplane with the largest interval. It achieves nonlinear classification using different kernel functions. Currently, it is widely used in EEG-based authentication [67]. Keshishzadeh et al. [36] used AR to extract features from the resting-state EEG signals of 104 individuals with closed eyes. Then, they used the exponential normalization method to normalize the extracted features based on the augmented reality model in two steps. Next, the features were mapped to [0, 1] before classification to produce more stable and better results. Using the SVM classification model, the method achieved an accuracy of 97.43% [36]. Brigham and Kumar [47] used AR parameters in feature extraction and exploited an SVM model for classification and authentication on the imagined speech data sets consisting of six subjects. This method achieved an average accuracy of 99.76% [47]. Bashar et al. [42] firstly used a band-pass filter to preprocess the EEG signal to remove noise, and then, these signals were divided into linearly independent segments. Next, three feature extraction methods were applied, including the multiscale shape description (MSD), multiscale wavelet packet statistics (WPS), and multiscale wavelet packet energy statistics (WPES). These features were finally used to train a supervised error correction output code multiclass model (ECOC) using SVM classification. The model passed preliminary tests on nine EEG records of nine subjects and achieved an accuracy of 94.44%. Besides, single-class SVM such as support vector data description (SVDD) is also used for authentication. SVDD is a single-value classification algorithm that can distinguish between target samples and nontarget samples. In the Graz data set B of the 2008 BCI competition, Pham et al. [46] took the AR parameters and PSD components of the signal as features and used SVDD classification, achieving an accuracy of up to 99.9%.

*(3) Low-Rank Sparse Decomposition*. The low-rank sparse decomposition method is proposed by Kong et al. [65], and it is different from the traditional shallow classification method. The existing studies revealed that the original EEG is composed of inherent background EEG, task-consciousness EEG, and noise. In [65], the superposition principle of the background part and the task-consciousness part of the EEG was studied. Also, the inherent background EEG was used for authentication through the low-rank sparse decomposition of the EEG signal. Kong et al. [65] exploited the GoDec+ algorithm to decompose the low-rank sparseness of the EEG signal and extracted the low-rank background EEG signal from the raw EEG data. The background EEG signal subspace of each subject was constructed and assembled into a whole space, and the test samples were matched to achieve efficient classification. Based on this, the feasibility of extracting background EEG for authentication under different data sets and specific tasks was verified, and an accuracy of more than 95% was reached. For a data set containing different tasks, the highest accuracy reached 99.95%. Because the low-rank sparse decomposition method discards task-related signals, it can be used on the data sets for various tasks theoretically.

*(4) Other Classification*. Zeynali et al. [67] used five mental activity data sets of seven subjects (325 samples). Through discrete Fourier transform, discrete wavelet transform, AR modeling, and entropy feature extraction, the Bayesian network (BN) [68] can achieve an accuracy of 85.97% [67]. Wang et al. [24] proposed a graph feature analysis method with brain-connected network topology features and classified features based on the Mahalanobis distance. Compared with traditional univariate methods, the proposed graph-based method improved the stability of authentication effectively [24]. Gui et al. [17] used overall averaging and low-pass filtering to reduce the noise of EEG signals. Then, according to the five main frequency sub-bands of the EEG signal, wavelet packet decomposition (WPD) was exploited to extract the features of the EEG signal. Based on artificial neural networks (ANNs), the classification achieved an accuracy of about 90% [17]. Wu et al. [34] used visually evoked EEG signals based on self-face and non-self-face, and they compressed the multichannel EEG signal into a single-channel signal. Then, the logistic regression analysis was conducted to extract the time-domain features. Finally, the classification was performed with hierarchical discriminant component analysis (HDCA), achieving an accuracy of 91.46%. In addition to the commonly used shallow classification for EEG-based authentication, the learning vector quantization (LVQ) [62], k-nearest neighbors (KNN) [49, 55], hidden Markov model (HMM), random forest (RF) [60, 69], etc., are also used for classification.

Due to the complexity of collecting EEG signals, the differences between the brain regions that induce excitement and the tasks of EEG signals, the commonly used feature extraction methods have their limitations. For example, it is difficult for a feature extraction method and classification to get good results on all individuals. Shallow classifications can be used in combination to achieve better classification results. The shallow classification methods for EEG authentication are listed in Table 3.

#### 6.1.2. Deep Learning

Shallow classifications can usually achieve good classification results when the classification boundary is relatively clear. This requires that the feature set obtained by feature extraction has a high degree of discrimination. Although it is possible to design feature extraction and classification methods for the EEG signals of specific tasks, it is difficult to apply the methods to EEG signals of other tasks to have a good performance. The data processing of traditional machine learning is easy to lose information. Meanwhile, specific feature extraction methods generally require the professional knowledge of field professionals, and the extracted features are single and insufficient. Besides, most existing machine-learning methods focus on studying static data, which cannot classify time-varying EEG signals accurately.

Therefore, it is proposed to use deep learning models for decoding EEG signals because the models can effectively capture the high-dimensional feature representation of the signals and the potential relationship of internal features through the nonlinear deep structure. For EEG signals with complex information content and strong time-varying and inconspicuous features, it is possible to extract a deep dimension and significant feature representation. Maiorana et al. [57] used Siamese CNN to learn deep features for task-independent authentication. In addition, the deep learning models can perform on the original data directly without complex preprocessing and feature extraction processes. These models can be used for feature extraction, classifiers, or feature extraction firstly and classification then as needed.

*(1) Convolutional Neural Network (CNN)*. A deep learning network model generally has many layers, and more deep-level internal features can be obtained through a deeper structure. In this case, the structure of a deep learning network is complex with a large number of parameters. If the data required for deep learning are sufficient, the learning process will be long and complicated. CNN is a deep neural network with a convolution kernel. Because of the convolution kernel, the convolutional layer is partially connected to the previous layer and shares parameters, which greatly reduces the number of network parameters. CNN has been widely used in image recognition and other applications, and great success has been achieved. It is considered to be able to extract salient features with specific and possibly unknown internal structures. In recent years, CNN has also been used by researchers to analyze and decode EEG signals [34, 43, 67].

Mao et al. [50] directly input the raw EEG data of 100 subjects during fatigue driving into CNN. The CNN with fully connected layers can accurately identify subjects with an accuracy of 97% [50]. Das et al. [37] used CAR spatial filter to preprocess the collected EEG signals on the motor imaging data set consisting of the data of 40 healthy subjects to reduce the potential inappropriate EEG signal artifacts. Then, the obtained data were spectrally filtered to obtain the alpha frequency band that is considered to contain the most important information in the EEG signals of this task. After multiple EEG signals of the same stimulus were averaged, a CNN with four convolution layers, two maximum pooling layers, one ReLU, and one softmax loss layer was used, achieving an accuracy of 99.3% after 200 iterations [37]. Zhu et al. [70] proposed a residual multiscale spatiotemporal convolutional neural network RAMST-CNN for authentication to efficiently utilize different levels of information. Through different scales of convolution kernels, the time-domain and space-domain features of the data were learned. Meanwhile, through global average pooling, the number of network parameters was reduced. The model extracted deep-level features from the original EEG signal and achieved a stable accuracy of 99.96% on multiple data sets [70]. Chen proposed a new CNN (GSLT-CNN) with global spatial and local time filters. The GSLT-CNN was exploited to directly process the original time-locked RSVP EEG signal of 157 subjects, and it achieved an accuracy of 99%. Because CNN has a stronger ability to process images, the EEG signals can be converted into images for classification by CNN.

To solve the problem of large model parameters and insufficient training samples, Jin et al. [40] proposed a convolutional tensor neural network (CTNN). CTNN uses CNN to mine the local features of EEG signals in the time and space domains of the EEG, and it uses the CTNN with a depth-wise separable convolution mechanism to extract the local spatiotemporal characteristics of the brain image. Then, the tensor network (TN) was used to capture the multi-linear related information, and the local information was integrated into global information with limited parameters. Finally, a small number of parameters of the tensor neural network were used to fuse the local features of the convolution output for classification. The experimental results show that the CTNN has great performance advantages for the data sets with large numbers of individuals and a small number of individual samples [40].

CNN can also be used for feature extraction to effectively extract target features. Salem and Lachiri [41] believed that there are unique individual differences in the emotional state contained in the EEG signal. Based on this, they exploited deep learning methods such as CNN to extract neural features from the EEG signal. Meanwhile, the features were considered as a repeatable discriminant feature, and then, the polynomial kernel function SVM was used to classify the features. On the MANHOB-HCI VEP visually evoked data set, an accuracy of 99.99% can be achieved [41].

*(2) Recurrent Neural Network (RNN)*. Although CNN can extract spatial features well, EEG signals are highly time-varying and contain rich timing features. As one of the major characteristics of EEG signals, the temporal characteristic reflects the changes in brain activity over time when the subjects perform tasks. While CNN fails to deal with the time-series features, RNN has excellent time-dependent expression capabilities. Therefore, RNN has advantages in processing time-varying EEG signals [26]. Zhang et al. [26] first input the decomposed delta frequency band into an attention-based RNN structure that assigns different attention weights to different EEG signal channels according to their importance. Based on attention scores or through various machine-learning algorithms such as reinforcement learning, the attention mechanism automatically redistributes the weights according to the changes in the environmental factors to extract the most distinctive features. The attention score can be inferred from the input data and used as a weight to make the model focus on the part with a high attention score. Reinforcement learning has shown that it is possible to find the most valuable part through strategic search. Based on the features learned by the attention-based RNN, the boosting classification was adopted for authentication. The method was evaluated on three data sets (two self-collected data sets and one public data set), reaching the accuracy of 98.2%, 98.82%, and 99.89%, respectively [26]. Besides, RNN can be used in combination with CNN to effectively extract time-domain and spatial-domain features. Wilaiprasitporn et al. [71] proposed a combination of CNN and RNN deep learning model, where CNN was used to process spatial information of EEG signals and RNN was used to extract time information. Meanwhile, long- and short-term memory (CNN-LSTM) and gated recurrence unit (CNN-GRU) were used. On the emotion data set, DEAP, CNN-GRU, and CNN-LSTM can realize authentication in different emotional states, achieving an average accuracy of 99.90–100% [71].

The RNN-based variants, such as LSTM and GRU, are also commonly used to decode EEG signals. The spatial resolution of the EEG signal is not high, and it is difficult to be accurate to the level of a single neuron based on the number of existing electrodes. However, the spatial relationship and mutual influence between the electrodes represent the inner neural characteristics of the brain area when performing tasks. These features can be captured efficiently with LSTM. Sun et al. [72] proposed a new 1D convolutional LSTM network to extract the spatial and temporal characteristics of EEG signals. Compared with CNN and LSTM individual methods, the proposed method achieved a higher accuracy of 99.58% on the PhysioNet data set [72]. Kumar et al. [29] used a sequence classification based on a bidirectional long- and short-term memory neural network (BLSTM-NN) to model the recorded sequence on the visually evoked data of an online real-time system of 58 subjects for authentication. In the gamma band, the BLSTM-NN achieved an accuracy of 97.57% [29].

*(3) Graph Convolutional Neural Network (GCNN)*. GCNN extends the CNN to the graph domain. Wang et al. [73] proposed a model for recognizing the EEG biometric features of different tasks. The EEG signal map was estimated based on intra-frequency and cross-frequency functional connectivity, and the GCNN was exploited to automatically capture the deep internal structure representation of the EEG signal map. The GCNN can generate a graphical representation of EEG signals and automatically extract deep structural features from the dynamic EEG signals for authentication. Meanwhile, the functional coupling between signals was adopted to solve the problem that relies on univariate characteristics, which helps GCNN to extract internal structural features. Based on this, an average accuracy of 99.98% can be achieved on the PhysioNet data set [73].

Although the EEG-based authentication method of deep learning has great advantages, it can be seen that different deep learning models have been used due to the difference in data, such as a different number of electrode positions, different data dimensions, different features, and different frameworks. Also, deep learning requires a large amount of data for model training, so a poor generalization may be obtained on the data set of other tasks. The deep learning methods for EEG-based authentication are listed in Table 4.

Although deep learning performs very well, it also has the same problems as other deep learning applications. One is that it requires a lot of training data to train the model, and the other is that training requires a lot of time and cost, and even high-performance computers, the third is that the problem of overfitting is serious. It is difficult for a model to have good actual test performance in all databases or application scenarios. The fourth is poor migration. This is also the direction of deep learning. Researchers hope that a model can better solve most of the same problems.

### 6.2. EEG-Based Biometric Cryptosystem Authentication

The method of biometric classification treats the unique characteristics corresponding to each person as a category and distinguishes each individual through classification to achieve the purpose of authentication. This authentication process is actually the decoding process of EEG signals in the brain-computer interface. However, authentication based on feature classification is faced with some problems. For example, the features extracted from the fake EEG signals generated by the generative adversarial network (GAN) will cause a misclassification. Adversarial sample attacks can also lead to misclassification. It is unwise to rely solely on biometric features for scenarios with high security levels, especially when the biometric features are mature and have been widely used. Biometric cryptosystems are proposed. Researchers hope to combine biometrics and cryptographic technology to improve authentication security and effectively resist existing attack methods [74].

Cryptography is considered as the foundation of information security, the key is the core foundation of authentication in the cryptographic system, and the key security is the dependence of the cryptographic system. In a generic cryptographic system, the possession of the decrypting key is a sufficient evidence to establish user authenticity, but the key is not closely related to the user's identity, so it is easy to be stolen or lost. Whether it is a legitimate user or an attacker, as long as the key is possessed, it can be decrypted. Biometric effectively solves this problem, but it is not flexible enough. Once biometrics is stolen, they cannot be used forever. Therefore, the biometric cryptosystems are proposed because cryptographic technology and biometric technology have very good complementarity. Biometrics closely related to identity information provides a good identity dependence for cryptography, and cryptography can protect biometric data while protecting user privacy, or use biometric data as a source of key generation. Biometric cryptosystems can be divided into key combining biometric cryptosystems, key generation biometric cryptosystems, and key binding biometric cryptosystems [59].

Compared with the traditional biometric authentication technology, the biggest advantage of EEG-based biometric cryptosystem authentication is its revocability. As for the traditional biometric authentication technology, once the user's characteristic information is lost or stolen, the characteristic can no longer be used. As for the EEG-based authentication, because of the high dependence of EEG signals on tasks [6] and the sensitivity to mental states such as emotions, the tasks that induce EEG signals can be changed to extract the unique characteristics of individuals corresponding to the tasks to generate different keys to achieve biometric revocability. In comparison, it has higher flexibility than the classification authentication method, and its anti-attack ability is greatly improved due to its revocability. It is difficult for an attacker to guess what state the legitimate user was in when collecting EEG and what task was performed. Even if it can be guessed, the features extracted from the imitation EEG signal have individual differences. In addition, revocability can also be achieved through some algorithms in biometric cryptosystems, such as fuzzy vault or BioHash.

#### 6.2.1. Key Combining EEG-Based Biometric Cryptosystems

The simplest way to realize a biometric cryptosystem is to combine biometrics and cryptography. The user needs biometric authentication and key authentication to succeed [75]. Although this is more secure than a single authentication, it is obvious that the two have not been bound. The shortcomings of these two are not removed. The system will still be at risk of attack.

Bajwa and Dantu [76] first used traditional EEG biometric classification authentication and used the key generated by EEG for authentication in the second phase. The first data set is based on five mental activities by 7 subjects, and the second is based on three visually evoked tasks by 120 subjects. In the traditional EEG-based biometric classification stage, they obtained a mean subject classification of 98.46% and 91.05% for Data set I and Data set II, respectively, using SVM and BN. In the key generation phase, they use the same EEG as in the classification phase. According to the similarity score, the feature vector is selected with the highest degree of discrimination. Then, the feature vector was binarized. The average key generated from EEG biometric is 230 bits per activity. The length of the key can be changed by combining different activities. After an appropriate choice of features, the mean half total error rate for generating keys was 3.05% for Data set I and 4.53% for Data set II. The keys generated from EEG biometric have been verified by the NIST statistical suite of randomness tests. The average entropy for their system was 82 bits. If the user's biometric information is leaked, the task can be changed to achieve revocability.

#### 6.2.2. Key Generation EEG-Based Biometric Cryptosystems

One of the shortcomings of traditional cryptography keys is that they cannot be clearly associated with personal identities. By contrast, the keys generated by encoding the EEG signal characteristics directly correspond to personal identities. In recent years, the combination of traditional biometric statistics and cryptography has been proposed to generate cryptographic keys. This requires the extraction of unique and repeatable biological information from the biological features. These biological features are limited by the acquisition technology or environmental conditions, and they are essentially noisy. Besides, the signal itself tends to be different from the measured values of the same user. This difference is caused by the inherent natural inconsistency of human physiology or the behaviors exhibited by external influences, but the key requirements must be correct and repeatable. For example, the iris is considered to be the most accurate feature of the traditional biometric features, but the difference between two different images of the same iris may be as high as 30% [77]. Therefore, overcoming the internal differences in biometric features is the main challenge for the use of biometric features in cryptographic systems. Also, considering the issues such as the possible loss of biometric features, it is desired that the key can be changed to avoid key leakage and improve security so that the key can be used in different encryption and authentication scenarios. However, traditional biological features are usually unchangeable. For example, fingerprints and iris hardly change in a person's life, which does not satisfy the revocability. The concealment of the EEG signal and the difficulty of forgery can improve the security of the generated key. Besides, the randomness of the EEG signal, the high dependence on the spirit, and the time correlation [78] make the EEG signal in different tasks generate different keys.

The key technology of key generation EEG-based biometric cryptosystems is the use of EEG signals to generate a unique and repeatable key. Some researchers have proved that EEG signals can generate random numbers. And the EEG signals can also be used as pseudorandom number generators [79] to generate keys for encryption[80] or authentication [81]. The measured EEG biometrics has a high entropy across subjects. The amount of uncertainty in the key from an attacker's point of view is large. However, most of the research still stays at the stage of generating keys, and few studies use generated keys for authentication. However, this does not affect the feasibility and broad application prospects of EEG signals to generate authentication keys. The EEG signal for key generation is listed in Table 5.

Another research focus of biometric cryptosystems is to address the issue of how biometric-based key schemes should handle the variability in the biometric representation. To make the key generated by the EEG signal usable, repeatable features need to be extracted from the EEG signal to accurately generate the repeated key. Therefore, it is necessary to repeatedly generate sufficiently random keys for the same individual. Essentially, the researchers are looking for individual differences or task differences. To this end, they extract the largest possible difference characteristics between different individuals performing the same task and the smallest possible difference characteristics between the same individual performing the same task. To encode the different features, some cryptographic protocols can be adapted to make the authentication more secure than the classification authentication. According to the chaos theory, the underlying subsystem of a nervous system that generates EEG signals is considered a nonlinear dynamics system, so EEG signals are chaotic [86]. Based on the nonlinear and chaotic characteristics of EEG signals, the EEG signal can be transformed into a random binary sequence through mathematical transformation [78]. In this way, EEG signals can be considered to be different from time to time. To generate duplicate keys during the authentication phase, quantization method and fuzzy extractor are used to produce the same results with slightly different input signals.

*(1) Quantization Method*. The quantization method is another commonly used method for generating keys from EEG signals, and it sets a global threshold for each feature. Using the quantization method, the key can be obtained through the modulus, difference [87], or XOR between the user's measured value and the threshold. Ravi et al. [85] extracted the event-related EEG signals in 61 electrode channels that were evoked by a single stimulus from 10 subjects. Then, an elliptical finite impulse response bandpass filter was used to filter the EEG signal from 30 to 50 Hz. Next, the energy of the filtered EEG signal was calculated and divided by the total energy from all the channels. These values were then normalized, and the positive normalized values are converted to a binary digit 1, while the negative values are converted to a binary digit 0. Since each channel corresponds to a binary character, the 61 channels of electrode data generated a 62 bit key [85]. Nguyen et al. [30] first performed frequency band filtering on the EEG signal. Then, the filtered signal was divided into several subsegments, and the average power was estimated from the output of the power spectral density estimation of each subsegment. Next, a key for each band was generated. Subsequently, 32 channels were randomly selected from the GrazIIIa data set, and the random selection was repeated 10 times to obtain the result as the average power value. In this way, a 256 bit key was generated, and the randomness of the key was proved by the NIST test [30]. Akhila et al. [88] collected EEG signals through specific tasks, and they used PCA to extract relevant feature vectors to quantify the key generation [88]. Similar to the cryptography of iris images [77] and the cryptography of face images [89], the topographic map images of EEG were converted into binary 0 and 1 to generate the key, but the reliability of this method has not been verified yet.

It is worth mentioning that classification methods are used to generate keys. Tuiri et al. [84] extracted the features of the EEG signals of eight subjects. The features were classified by SVM. Then, the classified features generated a 230 bit key by binarization. The key was used to encrypt and decrypt the data between two users. In essence, authentication determines the identities of the communicating parties [84].

*(2) Fuzzy Extractor*. To handle the inconsistency of the generated keys that may be caused by the internal differences in the same individual, the fuzzy extractor is used to make the output results consistent with the input data with a certain deviation. The fuzzy extractor requires a high minimum entropy value of the biometric features. Although the EEG signals generated by the same individual performing the same task at different times are different, the fuzzy extractor can extract the same uniform random string from similar input features within the allowable range of differences.

He and Wang [81] exploited a fuzzy extractor to generate a key and an auxiliary random string for a given EEG signal feature. When the user is authenticated, this random string and the EEG signal feature of the user under the same task can be used. The fuzzy extractor generates the same key as before, realizing the repeatability and accuracy of the key. Using BAN logic, the proposed authentication scheme was proved to be effective and practical [81]. Singandhupe et al. [80] used a fuzzy extractor to generate a 128 bit AES encryption key from the EEG signal in the beta band.

#### 6.2.3. Key Binding EEG-Based Biometric Cryptosystems

This scheme is based on error correction code. Error correction code is used to recover mistaken key's bit. EEG biometrics will be firstly quantified and then bound with the key held by the user through some algorithms. This scheme is very flexible. Researchers can use mature schemes (e.g., fuzzy commitment and fuzzy vault) or design schemes independently for authentication. The key can be personally held by the user or generated by EEG. Nguyen et al. [30] developed a multi-threshold error correction technique that can handle EEG signals with individual internal differences. Meanwhile, a biometric template was designed, and the template requires a user password, a specific task-specific EEG signal feature set, and a random string. During authentication, predefined tasks were performed to generate EEG signals, and the Berg method was used to perform AR analysis on short data segments to obtain the average power spectral density characteristics of the signal. This feature is one of the features set in the template, which can increase the complexity of the attackers guessing the correct key and reduce the recognition error to a certain extent. After a user enters the password into the template, the password is combined with the key generated by the feature quantization and the random string for authentication [30].

This scheme is not only more secure, but also can effectively protect EEG biometric. If an attacker can access the stored templates, the security of the EEG biometrics used will be compromised. Therefore, it is very important to design a suitable template protection scheme to generate revocable biometrics. In this scheme, the key and EEG are bound as a secure template. When any of the key and EEG biometrics are potentially vulnerable, any one or all of them can be revoked. BioHash is a technology that binds random numbers and biometric feature vectors for authentication with revocability. The random numbers can also be a key. The threshold is set to quantify the inner product value of the biometric feature vector and the random matrix, and authentication is achieved by comparing the quantified value during registration and authentication. Revocability can be achieved by updating the random matrix or the EEG biometric features. It enhances interclass changes while maintaining intra-class changes. When dealing with high-dimensional and large amounts of data, it has a faster calculation speed than traditional algorithms and is widely used in various security systems, but as far as we know, this algorithm has not been applied to EEG biometric.

*(1) Fuzzy Commitment*. The difficulty of binding cryptography with biometrics is that the digital key is accurate, while biometric technology is fuzzy, and fuzzy commitment is the way to solve this problem. In general, biometric sequences are nonbinary, and to apply fuzzy commitment, quantization has to be done firstly. The key K is a binary EEG biometric feature vector corrected by an error check code. The use of the error correction code can correct the bit error caused by the inaccuracy input EEG biometric and overcome the variability of EEG biometric. In this way, even though subjects are performing the same required tasks in different scenarios and emotions, they can still be successfully authenticated. The user selects a secret message C. The difference vector is denoted between the key K generated from user's biometric and C as d. The encrypted message consisted of d and *y* = hash (C). When decrypted, the biometric template is used to decode. In this scheme, both the revocation of C and the EEG evoked by the different tasks can realize the revocability of the system.

By combing fuzzy commitments with the Bose–Chaudhuri–Hocquenghem (BCH) error correction codes, Damaševičius et al. [82] generated a 400 bit key from the covariance matrix of 42 subjects' EEG signals for authentication. The performance of the biometric cryptosystem is a true-positive rate (TPR) of 0.9974. Yang et al. [83] used the two MI activity EEG signals obtained by the C channel from 10 subjects, filtered them with CAR to remove the average potential of all electrodes, and further normalized them to obtain the original EEG data. Feature vectors are obtained with AR. They believed that the binary quantification of biometric features is a hard decision, which will lose features' details, so they use equiprobable quantization, which is a multilevel quantization scheme. The result was encoded into a binary string. They used the method of fuzzy commitment to achieve a performance of around EER = 1.87% with effective key size of 21 bits.

However, one of the major shortcomings of the fuzzy commitment scheme is that it requires the biometric representation correspondence that is obvious. For a desired code length, optimal error correction codes are hard to acquire. The use of error correction codes may increase the possibility of successful authentication of illegal users. Using this code, the attacker also would have true match.

*(2) Fuzzy Vault*. The fuzzy vault based on biometrics is the most classic practical scheme in the field of biometric cryptosystem. A fuzzy vault scheme is an improvement of fuzzy commitment. In the lock phase (registration phase), it uses secret information to construct a characteristic polynomial *p*(*x*), then forms a disordered set F of EEG biometric points, which is mapped to a real characteristic point set V through the characteristic polynomial, and then randomly adds hash points. It binds the user's EEG biometric and secret information to generate real points and generates a vault database by adding a large number of hash points. When unlocking (authentication stage), the matching biometric reconstruction polynomial is required to recover the secret information. If the EEG features do not match, then the polynomial cannot be reconstructed. This scheme is widely used in biometric template protection and authentication. The biometric template is used to construct a fuzzy vault, and the vault is revocable by updating the random matrix, making it difficult for an attacker to obtain the true point information in the vault through related attacks, improving the reliability of the system and the security of the biometric template.

Albermany and Baqer [90] choose the C3, CZ, and C4 three channels of the left-hand and right-hand MI data from BCI Competition 2008—Graz data set A as their EEG data set. EEG signals were filtered between 8 and 30 HZ. PSD estimation using the Welch method is calculated to extract features. Five of the features are selected and quantized as integers. They select characteristic polynomial *p*(*x*) = 5*X*^2^ + 2*X* + 1 to map feature value, using the tent chaff points as random points to build the vault. The true-positive rates are up to 99% among 9 subjects, and the false-negative rates are up to 9%.

The EEG-based biometric cryptosystems have higher security, but we have to admit that there is still relatively little research in this area, which will be the focus of future research. It also has certain limitations. Although it is theoretically believed that the EEG signals generated by different people performing the same task are different, that is, the extracted task-related features are individual differences. Therefore, the feature code can be used as a unique key for identity authentication. However, the experiments conducted by Chiu et al. [91] show that attackers can perform impersonation attacks by imitating the tasks of legitimate users [91]. This indicates that the relevant characteristics of the same task between different people are very similar, so the uniqueness of the keys generated by different people on a large data set still needs to be proved. In addition, the key must be accurate, and legitimate users who want to generate repeated and accurate keys face some problems. Every time the user wears the collection device, the position of the electrode may not be exactly the same as before, which may cause input deviation. Even if the user performs the same task, the user's current state, such as psychological conditions (e.g., horror, pressure) and physiological conditions (e.g., inebriation, cold), may also cause input deviations [75].

## 7. Existing Problems and Development Direction of the EEG-Based Authentication

### 7.1. Uniqueness

According to the existing research, the current data set is generally small and few subjects are involved in the data set (usually dozens of subjects). Although each subject can be distinguished well on the data set, the authentication accuracy decreases as the number of subjects increases [92], which may be related to the classification and feature extraction methods. Also, it may be because no experiment or theory has yet proved that EEG signals have enough distinctive features (e.g., fingerprint signatures are distinctive in 7 billion people) to distinguish humans from each other. Tangkraingkij et al. [93] showed that the accuracy of the system could even be reduced by as much as 9% by only adding 10 subjects. Although it is theoretically proven that each person's EEG signal is different, it remains a question whether the EEG signal can be used for authentication on a large scale or even in humans [93].

### 7.2. Stability

The state of the human brain changes all the time, and it is affected by cognitive ability and mental state. It is still uncertain whether the EEG signal is as stable as other biological characteristics. Das et al. [37] collected two different data sets at an interval of one week for the same group of subjects, and CNN was used for training and testing. The obtained results confirm the hypothesis that EEG data have permanent distinguishing characteristics, providing the foundation for the future use of brain signals in biometric authentication systems [37]. Ruiz-Blondet et al. [39] have shown that after six months, some individuals can still be accurately identified by the authentication system. The experiment conducted by Wu et al. [34] found that the EEG signal induced by the face is relatively stable after 30 days. However, the stability of the EEG signal for a longer period and the stability of the individual differences with people's cognition cannot be guaranteed. Therefore, stability still needs to be proved.

### 7.3. Data Enhancement

It is costly and difficult to collect high-quality EEG signals, but a large amount of data is essential to training models, especially deep learning models. The result of the small amount of data is the poor generalization ability of the model. Therefore, data enhancement can increase the training data by generating artificial data that are not included in the original data set but has the data distribution characteristics of the original data set. There is growing evidence that the synthetic data extracted from generative models can be used for data enhancement to improve the performance of later classification tasks [94]. Abdelfattah et al. [95] proposed a new GAN model to learn the deep statistical characteristics of EEG signals, and they used the generated samples to expand the data set to improve the performance of the classification model [95]. Hartmann et al. [96] demonstrated that it is possible to generate artificial brain electrical signals with a GAN. By the improved Wasserstein GAN training [97], GAN can be trained gradually to generate artificial signals stably. The generated artificial signals are similar to the single-channel EEG signals in the time and frequency domains [96]. Aznan et al. [98] exploited a limited amount of EEG data collected from different subjects to train modern neural-based generative models to generate supplementary synthetic EEG signal vectors. The generated vectors were then used to train SSVEP classifications. Extensive experimental analysis shows that the generated data can improve the classification of real-world EEG data that are obtained from multiple topics and recorded under various conditions and sessions. Also, the analysis indicates that the use of synthetic EEG data can improve the convergence speed of the classification model. In this way, only a smaller amount of real training data is needed [98]. At the same time, it is desired to find a general learning model that can achieve high authentication accuracy on different task data sets.

### 7.4. Security Issues

Unforgeability is one of the advantages of EEG signals. However, in recent years, with the development of GAN technology, non-real data can be artificially generated, such as images, sounds, and EEG signals. Some researchers have used GAN to generate fake EEG data. The generated fake EEG signal can be successfully recognized by the system as the real EEG signal. Piplani et al. [99] proved that the fake EEG signals generated by GAN can deceive the system to recognize the signals as real signals of legitimate users. To mitigate this security vulnerability, the researchers used fake data together with real data as training data to train the classification model to make it more robust to this attack, and the classification accuracy of the model is higher than before [99]. By adding adversarial disturbance, Zhang et al. [100] controlled the brain-computer interface typewriter to tamper the characters desired by the subject to the characters desired by the attacker. This shows that artificially increasing adversarial disturbances can make the original EEG signals have the characteristics expected by the attacker [100]. Adding some confrontational disturbances to the EEG signals of legitimate users or intruders may result in the legitimate users being unable to successfully authenticate or the intruders being able to successfully authenticate.

Besides, EEG signals may be stolen [14] because EEG signals contain a large amount of personal identification information. Much of the information is private information that people do not want others to know, such as age, gender, and disease cognitive ability. If the EEG signal acquisition channel is attacked and stolen, the user's original EEG signal may be stolen through the transmission channel [101]. In this case, the signal obtained can be used to attack the system directly. Although liveness testing can be used to verify whether it is a copied or forged EEG signal, there is currently no specific and feasible method available. Therefore, it is necessary to encrypt the transmission channel and the storage of EEG signals to prevent attackers from stealing the EEG signals of legitimate users. For the purpose of solving the defects such as low accuracy, high time complexity, or slow processing speed, Liu et al. [102] used the Paillier encryption algorithm to encrypt EEG data. The neural network is used for the classification and recognition of encrypted EEG data [102]. Meanwhile, the EEG signals stored locally should be encrypted to prevent attackers from stealing [103].

### 7.5. Multimodal

Compared with a single-modal biometric authentication system, a multimodal system can provide better security. In such a system, it is difficult for the attacker to forge more than one biological feature at the same time, thus increasing the security of system. A multimodal system that performs authentication by combing EEG signal characteristics and other authentication methods can improve the system's anti-attack ability effectively. When other biometric features are combined for authentication, the unique characteristics of the EEG signals make up for the security loopholes of other biometric features for authentication, thus improving the authentication security level.

Zhang et al. [26] exploited the dual authentication system based on EEG signals and gait signals to overcome the limitations of the traditional single-modal biometric authentication system, which improves the accuracy of biometric authentication and risk prevention to a greater extent. After the user signal is received, the system only takes 0.39 seconds to complete authentication, fully meeting the requirements of practical applications in terms of system delay [26]. Studies show that although electrooculogram artifacts can affect the authentication accuracy of EEG signals, electrooculogram signals also have individual differences. Thus, electrooculogram features and EEG features can be exploited by multimodal authentication systems effectively [34]. Kumar et al. [29] proposed a multimodal system that can simultaneously capture dynamic signatures and EEG signals to develop a mobile user authentication system. Dynamic signatures are widely used for authentication based on behavioral attributes. Using the bidirectional long- and short-term memory neural network (BLSTM-NN) classification, single-modal and multimodal methods have been proposed for authentication [29]. Klonovs et al. [104] used the EMOTIV EPOC EEG signal acquisition equipment and facial detection to ensure that subjects are in a relatively quiet state to collect ERP EEG signals. Also, mature technologies such as the RFID identity cards based on near-field communication (NFC) were adopted to achieve real-time mobile biology authentication [104]. Rahman et al. [69] combined EEG and keystroke dynamics at signal level to authentication. Their data set was created by acquiring both keystroke dynamics and EEG signals simultaneously from 10 users. An accuracy of 99.6% is achieved using random forest classifier. Moreno-Rodriguez et al. [105] fused voice and EEG using a mixed signal-level-decision-level fusion scheme, with HMM on the first classification stage and majority vote for the final classification result, considering 50 users. An accuracy of 83.43% is achieved with an 80% voice-20% EEG mixed signal.

The multimodal authentication system can effectively overcome the limitations of an authentication method and improve the security and anti-attack ability of the system. However, the fusion of multimodality also decreases the efficiency of authentication and increases the redundancy of the system.

### 7.6. User-Friendly

The data collection device for authentication should be portable [49] and has high fault tolerance and anti-interference ability. Also, it is usually required to be nonintrusive. The existing data collection methods are often task-based that require subjects to perform tasks for a long time to obtain enough data to train the model. For some subjects, the tasks may not be pleasant, which can lead to mental fatigue of the subjects and even conflict with the experiment itself. In this case, the application of the authentication technology based on EEG signals is severely limited. Therefore, it is necessary to design a reasonable, efficient, and user-friendly experimental paradigm. Another consequence of an unfriendly experimental paradigm is that the subjects may produce data that are not useful to the experiment or even seriously affect the experimental results due to psychological factors. Therefore, the EEG signals collected for specific tasks are not completely credible. If the subjects do not follow the tasks required by the experimental paradigm, the collected data will affect the accuracy of the experimental results significantly. Therefore, the emotional feedback of the subjects on the experimental paradigm should be considered to design tasks that users are interested in.

For signal acquisition, the cumbersome wearing and adjustment of acquisition equipment are also intolerable to the subjects. Researchers have achieved high-accuracy authentication for a few electrodes or even a single electrode [5]. In this case, it is convenient to collect signals from subjects [44], reducing the cost and the complexity of EEG signal analysis and decoding. By reducing the number of brain electrical channels, Zeynali and Seyedarabi [67] achieved a high performance and determined the best electrode arrangement for different mental activities. The neural network classification achieved an average accuracy of 97-98% for the single-channel authentication system with the best placement of brain activity electrodes. Chuang et al. [55] exploited single-channel EEG signals to achieve an authentication accuracy of 99%.

## 8. Conclusions

This review analyzes the physiological characteristics of EEG signals and demonstrates their effectiveness and advantages as an authentication feature. According to the authentication steps of EEG signals, different task collection paradigms of EEG signals are first introduced. Then, in terms of EEG signal decoding, the commonly used data preprocessing and feature extraction methods are analyzed. Similar to the frequency band selection, the choice of electrode position is also highly dependent on the stimulation task. Next, the authentication methods are summarized, including EEG-based biometric classification authentication and EEG-based biometric cryptosystem authentication. It is worth mentioning that researchers can use a combination of methods in different steps for authentication. The current research on biometric cryptosystems is relatively small, but this is a very valuable research direction. Finally, the problems existing in authentication are proposed, and the solutions and development directions are discussed.

We found that in the EEG-based biometric cryptosystems, whether they are key combining EEG-based biometric cryptosystems, key generation EEG-based biometric cryptosystems, or key binding EEG-based biometric cryptosystems, key generation from EEG biometric is involved. We consider using deep learning to extract deep features and use multilevel quantization to minimize the loss of feature details to generate cryptographically required keys in our future work. Once we have the key, we can implement biometric cryptosystems or use the key in other encryption scenarios.

## Figures and Tables

**Table 1:** Different frequency band characteristics of EEG signals [17].

Frequency band	Frequency range	Amplitude	Status	Main region
Delta	1–4 Hz	20–200 uV	Infant, adult deep sleep, deep anesthesia, physiological coma	Occipital and frontal lobes
Theta	4–8 Hz	100–150 uV	Adolescents, adults in fatigue, depression, or old age	Parietal and frontal lobes
Alpha	8–13 Hz	20–l00 uV	Adults are sober and quiet	Posterior occipital lobe and parietal lobe
Beta	13–30 Hz	5–20 uV	Fully awake, irritated, and excited	Frontal and temporal lobes, and central areas
Gamma	>30 Hz	<2 uV	Cognitive learning and information processing	Somatosensory centre

**Table 2:** Number and positions of electrodes selected by different researchers for different tasks.

Researchers	Tasks	Numbers	Positions
Wu et al. [34]	ERP	16	Fz, Cz, P3, Pz, P4, Po7, Oz, Po8, C3, C4, F3, F4, Af7, Af8, Cp5, Cp6
Koike-Akino et al. [35]	ERP	14	AF3, AF4, F3, F4, F7, F8, FC5, FC6, P7, P8, T7, T8, O1, O2
Keshishzadeh et al. [36]	Resting state	6	C3, C4, P7, P8, O1, O2
Thomas et al. [31]	MI	19	Fp1, Fp2, F3, F4, Fz, F7, F8, T7, T8, C3, Cz, C4, P3, Pz, P4, P7, P8, O1, O2
Gui et al. [17]	VEP	6	Fpz, Cz, Pz, O1, O2, Oz
Das et al. [37]	MI	17	FZ, F3, F4, F7, F8, CZ, C3, C4, T3, T4, PZ, P3, P4, T5, T6, O1, O2
Kumar et al. [29]	VEP	14	AF3, AF4, F3, F4, F7, F8, FC5, FC6, P7, P8, T7, T8, O1, O2
Huang et al. [38]	VEP + sound	14	AF3, AF4, F3, F4, F7, F8, FC5, FC6, T7, T8, P7, P8, O1, O2

**Table 3:** Shallow classification methods for EEG authentication.

Researchers	Tasks	Feature extraction	Classification	Accuracy (%)
Kong et al. [65]	MI	Node degree of brain network	LDA	99.1
	DRI mental task			99.3

Salem et al. [41]	MANHOB-HCI VEP	CNN	SVM	99.99
Seha et al. [52]	Listening	CCA	LDA	96.46
Wu et al. [34]	FRSVP VEP	Fisher LDA and logistic regression	HDCA	91.46
Koike-Akino et al. [35]	ERP	PCA and partial least squares	LDA	96.70
Brigham et al. [47]	Imagined speech	AR	SVM	99.76
Jayarathne et al. [66]	Listening + VEP + ERP	CSP	LDA	96.97
Keshishzadeh et al. [51]	Resting state	AR	SVM	97.43
Thomas et al. [31]	Resting state	Individual alpha frequency (IAF) delta band power (DBP)	Cross-correlation values and Mahalanobis distance	90
Bashar et al. [42]	Resting state	MSD, WPES	ECOC-SVM	94.44
Gui et al. [17]	VEP	WPD	ANN	90
Pham et al. [46]	MI	AR, PSD	SVDD	99.90
Zeynali et al. [67]	Mental task	DFT, DWT, AR	BN	85.97
			SVM	84.49

Wu et al. [34]	RSVP	Fisher LDA	HDCA with genetic algorithm	94.26

**Table 4:** Deep learning methods for EEG-based authentication.

Research	Tasks	Number of subjects	Number of electrodes	Deep learning model	Accuracy (%)
Sun et al. [72]	Resting state	109	16	LSTM	99.58
Mao et al. [50]	ERP	100	64	CNN	97.00
Wang et al. [24]	Resting state	109	64	GCNN	99.98
Das et al. [37]	MI	40	17	CNN	99.30
Wilaiprasitporn et al. [71]	ERP with emotion			CNN ＋ RNN	99.90
Chen et al. [58]	ERP	100		GSLT-CNN	97
	RSVP	10			99
	ERP with emotion	32			99
	Multiple data sets	157			96

Zhang et al. [26]	Resting state	8	14	Attention-based RNN	98.20
	MI	8	64		99.89

Wu et al. [34]	RSVP with eye blinking	10	16	CNN	97.60
Kumar et al. [29]	VEP	58	14	LSTM	97.57
Wang et al. [73]	SSVEP	10	8	CNN	99.73

**Table 5:** EEG signal generation key.

Researchers	Methods	Key length (bits)	Number of subjects
Singandhupe et al. [80]	Fuzzy extractor	128	
Damaševičius et al. [82]	Fuzzy commitment	400	42
Yang et al. [83]	Fuzzy commitment	21	10
Tuiri et al. [84]	Quantization	230	8
Bajwa and Dantu et al. [76]	Quantization	230	120
Nguyen et al. [30]	Quantization	256	3
Ravi et al. [85]	Quantization	62	10
